# The impact of ambidextrous leadership on innovative work behavior among critical care nurses: a cross-sectional study

**DOI:** 10.1186/s12912-025-04232-0

**Published:** 2025-12-29

**Authors:** Ahmed Abdelwahab Ibrahim El-Sayed, Doaa Fawzy Hassan Ghazy, Gehan Galal Elbialy, Nadia Hassan Ali Awad, Heba Mohamed Al-anwer Ali Ashour

**Affiliations:** 1 Nursing Administration Department, Faculty of Nursing, Alexandria University, 9 Edmond Vermont Street - Smouha, Alexandria, Egypt; 2 Nursing Program, Batterjee Medical Collage, Jeddah, 21442 Saudi Arabia; 3 College of Nursing - Jeddah, King Saud bin Abdul-Aziz University for Health Sciences, Jeddah, Saudi Arabia

**Keywords:** Ambidextrous leadership, Critical care nurses, Innovative work behavior

## Abstract

**Background:**

Innovation is vital in high-acuity healthcare settings, yet engagement in innovative work behavior (IWB) among nurses remains limited. Ambidextrous leadership, which integrates explorative and exploitative styles, may enhance nurses’ innovation capacity.

**Aim:**

To examine the influence of ambidextrous leadership behaviors on innovative work behavior among critical care nurses.

**Methods:**

A cross-sectional correlational study was conducted with 360 nurses in critical care units at a major educational hospital in Egypt. Data was collected over 2 months in 2024 using the Ambidextrous Leadership Questionnaire and the Innovative Behavior Inventory. Descriptive statistics, Pearson correlation, and regression analyses were used to assess relationships between study variables.

**Results:**

The mean score for nurses’ perception of ambidextrous leadership was 40.10 ± 5.69, and for innovative work behavior was 66.03 ± 8.35. Both values reflect moderate levels. Ambidextrous leadership was positively correlated with IWB (*r* = 0.483, *p* < 0.001). Exploitative leadership showed a stronger correlation with IWB (*r* = 0.466) compared with explorative leadership (*r* = 0.381). Regression analysis further confirmed that ambidextrous leadership significantly predicted IWB (β = 0.304, R² = 0.625, *p* < 0.001).

**Conclusion:**

Ambidextrous leadership is a significant driver of innovative behavior among critical care nurses. By balancing creativity with clinical discipline, nurse leaders can foster innovation without compromising care quality. These findings highlight the importance of developing ambidextrous leadership skills to promote innovation in critical care practice.

**Clinical trial number:**

Not applicable.

**Supplementary Information:**

The online version contains supplementary material available at 10.1186/s12912-025-04232-0.

## Introduction

Innovation is increasingly essential in modern nursing practice, particularly in high-acuity environments where complex clinical demands require adaptive and creative solutions. Nurses are expected not only to provide safe, high-quality care but also to drive improvements in service delivery. However, recent research indicates that levels of innovative work behavior (IWB) among nurses remain low, especially in resource-constrained settings [1, 2]. Ambidextrous leadership may play a critical role in fostering innovation. Although it has been linked to improved performance and innovation in other sectors, its impact on nurses remains underexplored [3–6].

## Theoretical framework

This study draws upon Organizational Ambidexterity Theory (March, 1991) [7] and Leader–Member Exchange (LMX) Theory to frame the relationship between leadership and IWB in nursing. Organizational Ambidexterity Theory postulates that organizations must balance exploration and exploitation to remain adaptive and sustainable. Applied to leadership, this translates into behaviors that alternately “open” space for creativity and “close” it with direction and discipline. Explorative leadership includes encouraging new ideas, tolerating mistakes, and creating psychological safety for experimentation. In contrast, exploitative leadership emphasizes monitoring, adherence to standards, and performance accountability [7]. The effective integration of these behaviors enables ambidextrous leaders to harness creativity without undermining reliability, which is particularly important in patient safety–critical contexts like intensive and critical care nursing [8].

**Fig. 1:**
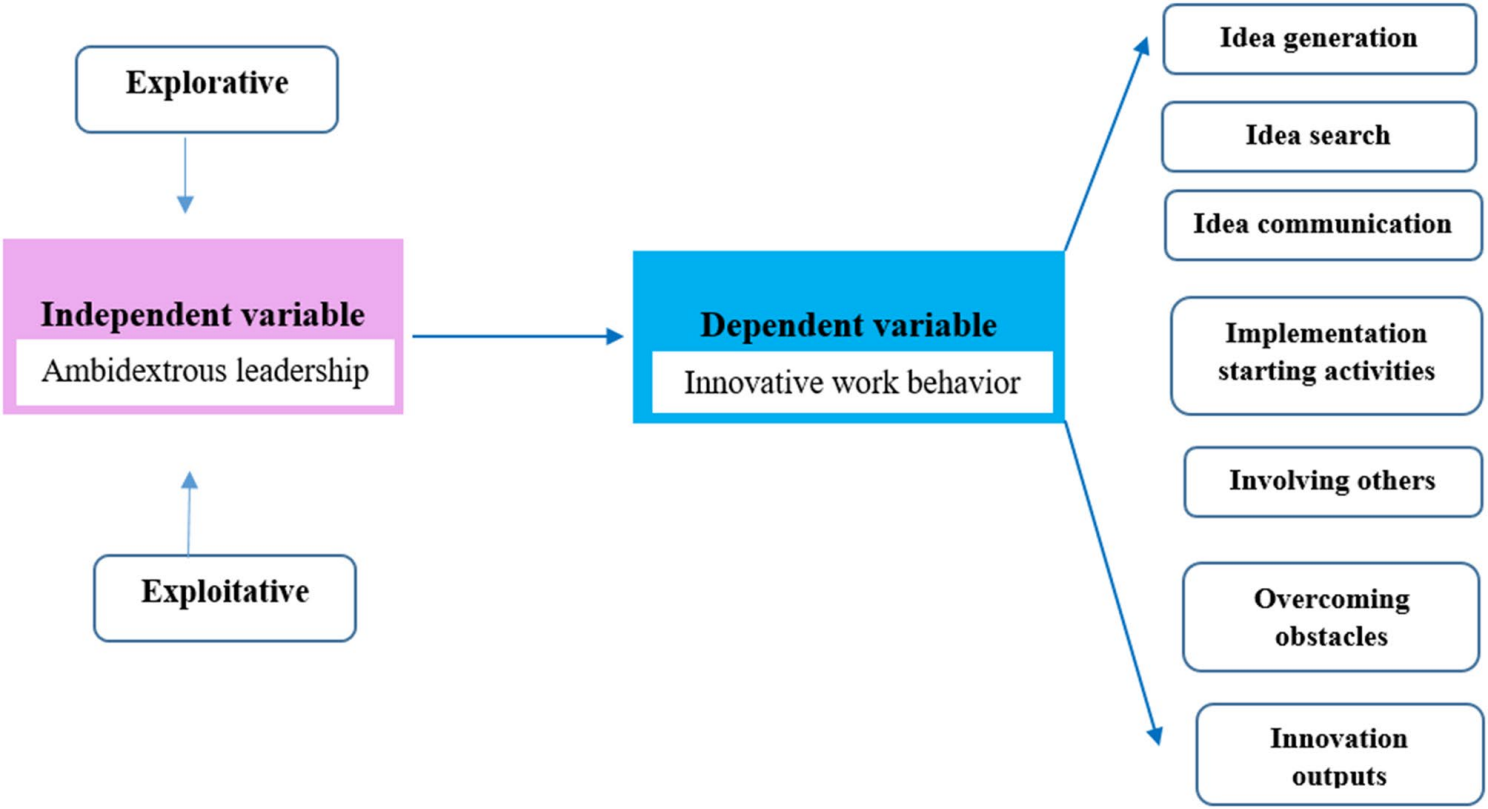
Conceptual framework for the study of ambidextrous leadership and innovative work behavior among critical care nurses

LMX Theory complements this by emphasizing the quality of the dyadic relationship between leader and follower. Leaders who build high-quality relationships characterized by trust, respect, and open communication can create conditions for IWB to flourish [9]. When nurses perceive strong LMX, they are more likely to share ideas, engage in problem-solving, and experiment with innovative practices. Thus, combining AL with LMX provides a dual lens: ambidextrous leadership offers the structural mechanisms for balancing innovation and discipline, while LMX highlights the relational mechanisms that empower nurses to act on innovative impulses [10]. By synthesizing these frameworks, the present study positions AL not as a theoretical abstraction but as a practical, relationally grounded style of leadership that can meaningfully shape IWB in high-pressure nursing contexts. The conceptual model is illustrated in Fig. 1.

## Background

Ambidextrous leadership refers to a dynamic leadership style that alternates between two distinct yet complementary behaviors: explorative (opening) and exploitative (closing) leadership. Explorative behavior, or “opening” behavior, involves encouraging team members to question existing routines, engage in creative thinking, and seek novel solutions [3]. Explorative leaders create an environment that rewards curiosity, risk-taking, and proactive problem-solving [4]. Jabeen et al. (2023) highlighted that explorative leadership fosters psychological safety and empowers nurses to share innovative ideas without fear of criticism or failure (11).

Otherwise, exploitative, or “closing” behavior, emphasizes adherence to established protocols, standardization, and process efficiency [4]. In clinical settings, this includes ensuring compliance with infection control protocols, medication administration guidelines, and quality assurance mechanisms [8]. Cai et al. (2023) illustrated that exploitative leadership is essential in integrating innovation into routine care by reducing variability and aligning new practices with existing systems [12]. For instance, nurse leaders who emphasize exploitative behavior may streamline shift handover procedures to reduce communication errors, thereby operationalizing new ideas while safeguarding clinical standards [4].

This dynamic balance between explorative and exploitative behaviors forms the foundation of ambidextrous leadership [13]. Leaders who can seamlessly shift between these approaches are more capable of managing current demands while also promoting sustainable innovation and future development [14]. As highlighted by Mutonyi et al. (2024), achieving this balance is particularly vital in critical care environments, where the pursuit of innovation must align with the essential requirements of accuracy and consistency [15].

IWB is a multidimensional concept encompassing all behaviors associated with the intentional creation, promotion, and implementation of new ideas in the workplace to improve outcomes [16]. In the nursing context, IWB involves identifying gaps in care, suggesting new practices, and facilitating their integration into care delivery. According to Lukes and Stephan (2017), IWB encompasses seven interrelated dimensions: idea generation, idea search, idea communication, implementation starting activities, involving others, overcoming obstacles, and innovation outputs, that collectively represent the process of translating novel ideas into practice [17].

Idea generation involves creating original and context-relevant solutions aimed at improving workplace processes or outcomes. Idea search refers to the active pursuit of information, evidence, or expert input to refine and support these emerging ideas [18]. Once an idea has been developed, idea communication becomes critical; this involves clearly articulating and advocating for it to colleagues or supervisors to gain feedback, collaboration, or approval. Implementation starting activities include taking practical steps to initiate the innovation process, such as planning, allocating resources, or initiating pilot trials [19]. Involving others refers to the collaborative aspect of innovation, wherein team members or stakeholders are engaged to contribute to or support the innovation effort. Overcoming obstacles reflects the persistence and adaptability required to navigate barriers or resistance during the implementation phase [20]. Finally, innovation outputs denote the successful realization of ideas in the form of improved services, processes, or patient care outcomes [17].

Jabeen et al. (2023) emphasized that IWB in nurses goes beyond mere ideation and must include effective execution and collaboration with multidisciplinary teams [11]. Similarly, Abd-Elmoghith et al. (2024) reported that IWB contributes not only to individual job satisfaction but also to organizational performance and patient outcomes [21]. Studies have increasingly demonstrated that ambidextrous leadership is positively associated with IWB in healthcare, although most evidence comes from non-nursing contexts [5, 11, 16].

## Research gap and significance of the study

The global health sector is under immense pressure to deliver high-quality, cost-effective, and innovative care. Nurses, who form the largest segment of the healthcare workforce, are central to these reform efforts [10]. In particular, IWB among nurses is vital for improving patient outcomes, enhancing safety, and optimizing workflow efficiency [11]. However, despite its importance, empirical evidence shows that nurses often report moderate or low engagement in IWB across different healthcare systems [12].

In Egypt, this challenge is magnified by systemic and organizational constraints. Egyptian nurses frequently work in environments characterized by limited resources, hierarchical structures, and variable staffing ratios [22]. These conditions can inhibit the free expression of new ideas and the adoption of innovative practices [13]. Moreover, critical care units (CCUs) in Egypt are particularly demanding, with high patient acuity and constant exposure to life-threatening conditions. Nurses working in these units must make rapid, high-stakes decisions, which can create tension between adhering to established protocols and experimenting with novel approaches. Leadership thus becomes a critical factor in determining whether innovation is stifled or supported [23].

Although research on nursing leadership in Egypt has expanded in recent years, most studies have focused on transformational and transactional leadership styles, with limited attention to ambidextrous leadership. The few existing studies in Middle Eastern and low- and middle-income contexts suggest that leadership practices often lean heavily toward control and discipline, with limited room for exploration and creativity [5, 15]. This imbalance may suppress IWB, leaving a significant gap in both the literature and practice. Therefore, examining AL in the Egyptian critical care context is not merely a matter of geographic interest; it directly addresses the broader research gap concerning how leadership can enable innovation in resource-constrained systems.

Egypt also provides an important case study because of its ongoing healthcare reform efforts aligned with Egypt’s Vision 2030. These reforms emphasize quality improvement, workforce development, and the integration of innovative practices into healthcare delivery [22]. Investigating how AL shapes nurses’ IWB in this context offers insights that are both locally relevant and globally transferable, particularly for other countries with similar systemic constraints.

## Methods

### Aim of the study

This study aimed to examine the influence of explorative and exploitative behaviors of ambidextrous leadership on innovative work behavior among critical care nurses.

### Research question

How do explorative and exploitative behaviors of ambidextrous leadership influence innovative work behavior among critical care nurses?

### Research design and setting

A cross-sectional correlational research design was employed in this study, as it is well-suited for examining the relationships between variables without manipulating them. The study was carried out across all CCUs (*N* = 23) within the Main University Hospital affiliated to Alexandria University in Egypt. This hospital is the largest academic medical center in the Alexandria Governorate, offering free public healthcare services, and serves a broad segment of the population.

### Study participants

The target population for this study comprised all registered nurses working in CCUs in a governmental hospital in Alexandria University, Egypt. The accessible population included nurses who were available and eligible to participate at the time of data collection in selected CCUs. A non-probability convenience sampling technique was employed to recruit 360 critical care nurses due to the practical constraints of accessing a dispersed and high-demand clinical population. While this approach allows for efficient data collection within resource and time limitations, it may limit the generalizability of the findings beyond the sampled population. Participants were eligible for inclusion if they met the following criteria: provided either direct or indirect patient care in CCUs; voluntarily agreed to participate in the study; and had a minimum of six months of clinical experience in their current workplace, ensuring sufficient familiarity with institutional roles, policies, and procedures.

The minimum required sample size was calculated using G*Power version 3.1 for multiple regression analysis with a medium effect size (f² = 0.15), power (1 - β) of 0.95, and an alpha level of 0.05. Based on 10 predictor variables included in the regression model, the minimum sample size required was 172 participants. To enhance statistical power and account for potential missing data or incomplete responses, a final sample of 360 nurses was recruited.

### Study instruments

Three tools were used in the study to collect the required data.

#### Tool I: Demographic and work-related data

This tool was created by researchers to gather data on participants’ demographic and professional backgrounds. It included items addressing nurses’ age, gender, educational level, years of professional experience, current work unit, marital status, and prior participation in training programs related to leadership.

#### Tool II: Ambidextrous leadership questionnaire (ALQ)

This tool was designed by Rosing et al. (2011), to evaluate first-line nurse managers’ ambidextrous leadership behaviors as perceived by nurses [3]. It consists of 14 items divided equally into two dimensions: explorative behaviors and exploitative behaviors. Each item is rated on a five-point Likert scale ranging from 1 (strongly disagree) to 5 (strongly agree). Therefore, the score for each subscale ranges from 7 to 35, and the overall ambidextrous leadership score ranges from 14 to 70. Based on total scores, leadership behaviors levels can be interpreted as low (14–32), moderate (33–51), or high (52–70). In its original validation, the tool demonstrated strong internal consistency, with Cronbach’s alpha coefficients of 0.90 for exploitative behaviors and 0.81 for explorative behaviors [3].

#### Tool III: Innovative behavior inventory questionnaire (IBIQ)

This tool was developed by Lukes and Stephan (2017) [17], to assess the level of innovative work behavior among nurses. It comprises 23 items distributed across seven dimensions: idea generation (3 items), idea search (3 items), idea communication (4 items), implementation starting activities (3 items), involving others (3 items), overcoming obstacles (4 items), and innovation outputs (3 items). Each item is rated on a five-point Likert scale ranging from 1 (strongly disagree) to 5 (strongly agree), resulting in a total score range of 23 to 115. Based on total scores, innovative work behavior can be categorized as low (23–53), moderate (54–84), or high (85–115). The tool demonstrated high reliability, with a Cronbach’s alpha coefficient of 0.864 [17].

### Tools adaptation, validity, and reliability

#### Translation and cultural adaptation

The instruments underwent Arabic translation to align with the Egyptian cultural context. A back-translation method was implemented by bilingual experts to preserve both semantic and conceptual consistency. This rigorous translation process guaranteed that the tools were linguistically accurate and culturally suitable for use by Egyptian nursing professionals. The tools were used and translated for academic research purposes based on publicly available versions, with full credit to the source.

#### Validity assessment

**Fig. 2:**
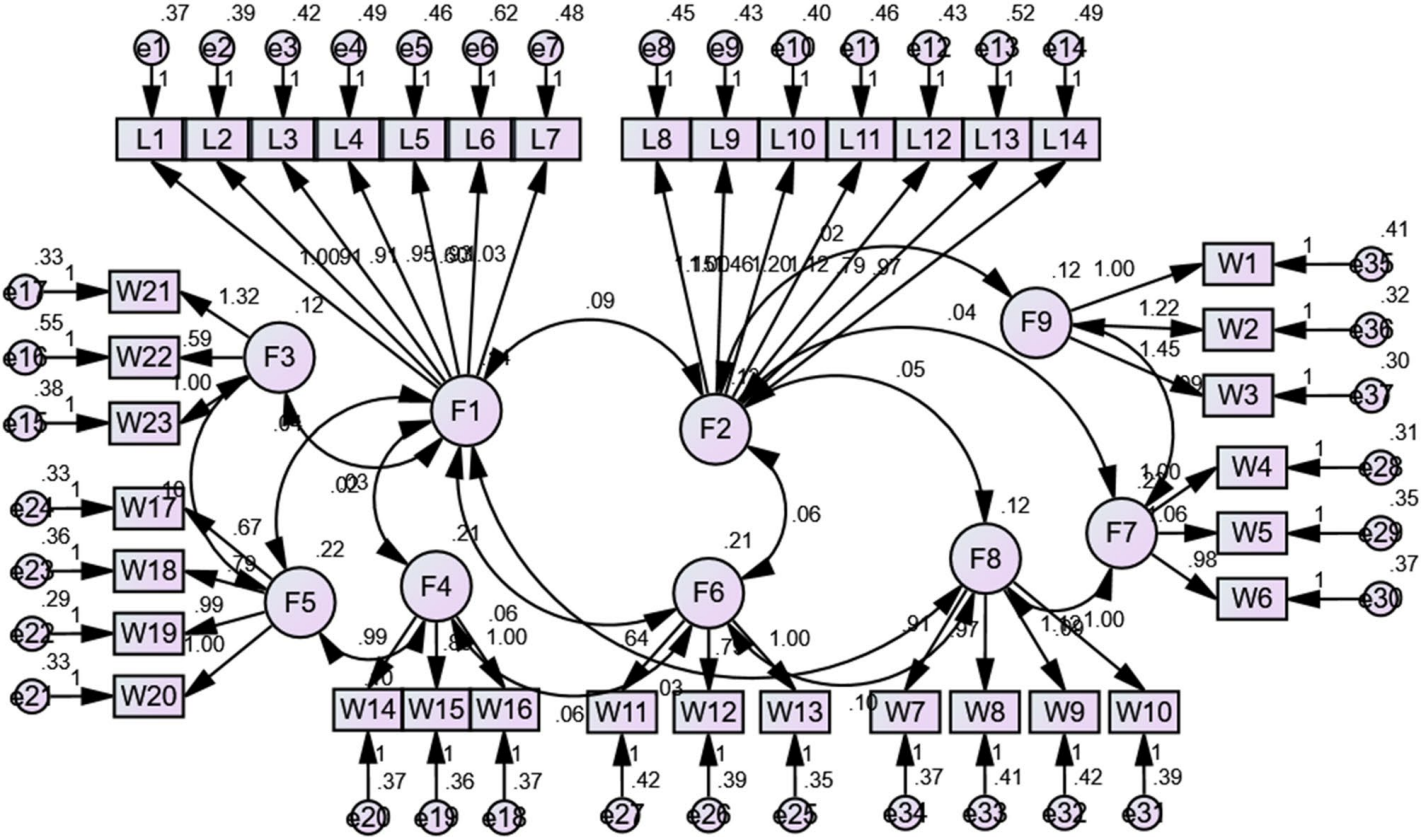
Confirmatory factor analysis (CFA) using structural equation modeling (SEM) between explorative and exploitative ambidextrous leadership and innovative work behavior. The model fit indices: Comparative Fit Index (CFI); Incremental Fit Index (IFI); Root Mean Square Error of Approximation (RMSEA); χ². CFI=1.000; IFI= 1.000; RMSEA=0.054; χ² = 2.033 (p = 0.001)

The Arabic versions of the instruments were subjected to rigorous validity assessment. Content and face validity were evaluated by a panel of five academic experts in nursing administration from Alexandria University, who reviewed the tools for clarity, relevance, and cultural appropriateness. Based on their feedback, minor linguistic adjustments were made. The overall Scale Content Validity Index (S-CVI) was 0.876, and all Item-Level Content Validity Index (I-CVI) scores exceeded 0.80, indicating acceptable content validity. Additionally, construct validity was assessed using Confirmatory Factor Analysis (CFA) through structural equation modeling (SEM). The model fit indices were satisfactory, with a Comparative Fit Index (CFI) and Incremental Fit Index (IFI) of 1.000, Root Mean Square Error of Approximation (RMSEA) of 0.054, and χ² = 2.033 (*p* = 0.001), confirming the validity of the factor structure in the translated versions for the Egyptian context, *see* Fig. 2.

Standardized factor loadings from the CFA model indicated strong convergent validity. All loadings exceeded the recommended threshold of 0.60 and ranged from 0.66 to 0.89. Specifically, for the Ambidextrous Leadership construct, the standardized loadings ranged from 0.68 to 0.88 for explorative behaviors and 0.66 to 0.87 for exploitative behaviors. For the Innovative Work Behavior construct, factor loadings across the seven dimensions ranged from 0.70 to 0.89. All standardized loadings were statistically significant (*p* < 0.001), confirming the robustness of the measurement model, see supplementary file Table 1s.

#### Pilot testing

A pilot study was conducted on 10% of the total sample (*n* = 36) from Alexandria Main University Hospital to evaluate the clarity, applicability, and feasibility of the study instruments. The pilot aimed to identify potential challenges in data collection and to estimate the time needed to complete the questionnaires. The findings indicated that the tools were clear and practical, and no modifications were deemed necessary. Participants in the pilot study were excluded from the main study sample to avoid potential bias.

#### Reliability analysis

Internal consistency was assessed using Cronbach’s alpha based on the current study sample. The ALQ showed a Cronbach’s alpha of 0.720, while the IBIQ demonstrated a reliability coefficient of 0.857, indicating satisfactory internal consistency for both tools in the local context.

### Data collection

Data was collected from the staff nurses through hand-delivered questionnaires to study subjects in their work settings from different categories in the morning, evening, and night shifts. After an individualized interview with each nurse for about 10 min to explain the aim of the study, the needed instructions were given. They were given some general instructions regarding how to respond to questionnaires. Then the specific instructions about each questionnaire were also made clear to them. They were requested to read each statement carefully and select the appropriate response option that they think well represents them. Each nurse took from 15 to 20 min to fill out the questionnaires. Data collection took two months, from the beginning of January 2024 to the end of February 2024. Before analysis, all returned questionnaires were reviewed for completeness. Questionnaires with more than 10% missing data were excluded from the analysis, while those with minimal missing values were included after applying mean imputation for the missing items within the same subscale.

### Ethical considerations

The study received approval from the Ethics of Research Committee at the Faculty of Nursing, Alexandria University (IRB00013620, SN:13-9-2023). Administrative permission was also obtained from the hospital prior to data collection. Written informed consent was secured from all participants after clearly explaining the study’s purpose, ensuring confidentiality, anonymity, and privacy. Participants were assured of their right to voluntarily participate and withdraw from the study at any time without any consequences.

### Statistical analysis

Data were analysed using IBM SPSS Statistics version 25 and AMOS version 23.0. Descriptive statistics were utilized to summarize the data. Continuous variables were described using minimum, maximum, mean, and standard deviation, while categorical variables were presented as frequencies and percentages. To evaluate responses from the study instruments, composite scores were computed by averaging the sum of the individual item scores for each scale. Both the Ambidextrous Leadership Questionnaire (ALQ) and the Innovative Behaviors Inventory Questionnaire (IBIQ) produced composite scores ranging from 1 to 5. For analytical purposes, composite scores were categorized into three levels based on their distribution: scores falling within the lowest third (i.e., below the 33%) were classified as low, scores within the middle third (33–66%) as moderate, and those in the highest third (above 66%) as high [20]. This categorization was used only to enhance interpretability of the results and was based on dividing the theoretical range of each scale into three equal intervals, a widely used approach in scale-based nursing and leadership research (e.g., Tao et al., 2025; Cai et al., 2023) [10, 12]. Similar methods for categorizing Likert-type scale scores are supported in methodological literature [24–26].

Inferential statistical analyses were conducted to examine relationships among the study variables. Pearson’s correlation coefficient (r) was used to assess the strength and direction of linear associations. Statistical significance was determined at a threshold of *p* ≤ 0.05. Additionally, hierarchical regression analysis was employed to assess the predictive power of ambidextrous leadership on nurses’ innovative work behavior, while controlling for the effects of demographic variables.

Structural Equation Modeling (SEM) was conducted using AMOS version 23.0 to test the hypothesized relationships between ambidextrous leadership and nurses’ innovative work behavior. The model was developed based on the theoretical framework and existing literature. Two latent variables: Explorative Leadership and Exploitative Leadership were specified as indicators of Ambidextrous Leadership, which was modeled as an exogenous (independent) construct. Innovative Work Behavior was treated as an endogenous (dependent) construct composed of multiple observed indicators (e.g., idea generation, idea promotion, idea realization).

Model specification included the identification of paths between ambidextrous leadership and each component of innovative behavior. Maximum Likelihood Estimation (MLE) was used for model estimation. Model fit was assessed using multiple goodness-of-fit indices: Chi-square (χ²), the Comparative Fit Index (CFI), Incremental Fit Index (IFI), and the Root Mean Square Error of Approximation (RMSEA. Acceptable model fit was indicated by CFI and IFI values ≥ 0.90, RMSEA ≤ 0.08 [27].

## Results

**Table 1:** Demographic and work-related data of study subjects (*n* = 360)

Variables	Category	*N*	%
**Gender**	Male	106	29%
	Female	254	71%
**Age (years)**	< 30	131	36%
	30–39	129	36%
	40–49	77	21%
	≥ 50	23	6%
	*Range*: 21–59	*Mean ± SD*: 34.1 ± 8.1	
**Years of nursing experience**	< 5	73	20%
	5–9	107	30%
	10–14	44	12%
	15–19	34	9%
	≥ 20	102	28%
	*Range*: 1–40	*Mean ± SD*: 13.0 ± 9.6	
**Years of experience in current department**	< 5	81	23%
	5–9	107	30%
	10–14	40	11%
	15–19	34	9%
	≥ 20	98	27%
	*Range*: 1–40	*Mean ± SD*: 12.6 ± 9.6	
**Qualification**	Bachelor’s degree	168	47%
	Associate degree	55	15%
	Diploma	137	38%
**Marital status**	Married	219	61%
	Single	139	39%
	Widow	2	1%
**Attended training on leadership styles**	Yes	3	1%
	No	357	99%
**Studied courses on leadership styles**	Yes	13	4%
	No	347	96%

Table 1 presents the demographic and work-related characteristics of the study participants (*n* = 360). The sample consisted predominantly of female nurses (70.6%), with males representing 29.4%. The participants were relatively young, with 36.4% under the age of 30, and 35.8% aged between 30 and < 40 years. The mean age was 34.12 ± 8.10 years. Regarding professional experience, 29.7% of nurses had 5 to < 10 years of experience, and 28.3% had ≥ 20 years. The average duration of experience in the nursing profession was 12.98 ± 9.61 years. A similar pattern was observed in departmental tenure, with a mean of 12.59 ± 9.58 years, suggesting that most nurses had significant exposure to critical care settings. In terms of educational attainment, 46.7% of the nurses held a bachelor’s degree, while 38.1% held a diploma, and 15.3% had an associate degree. Regarding marital status, 60.8% of participants were married, 38.6% were single, and 0.6% were widowed. Alarmingly, only 0.8% of nurses reported attending any training related to leadership styles, and just 3.6% studied courses related to leadership.

**Table 2:** Descriptive statistics for ambidextrous leadership behaviors and innovative work behavior from nurses’ perspectives (*n* = 360)

Study variables	Total score			Average Score (1–5)
	Score Range	Min. – Max.	Mean ± SD.	Mean ± SD.
**Ambidextrous Leadership (ALQ)**				
Explorative leadership behaviors	(7–35)	10.0–30.0	20.21 ± 3.05	2.89 ± 0.44
Exploitative leadership behaviors	(7–35)	7.0–31.0	19.89 ± 3.41	2.84 ± 0.49
**Overall**	(14–70)	19.0–60.0	40.10 ± 5.69	2.86 ± 0.41
**Innovative Behavior Inventory (IBIQ)**				
Idea generation	(3–15)	4.0–14.0	8.84 ± 1.62	2.95 ± 0.54
Idea search	(3–15)	4.0–15.0	8.86 ± 1.83	2.95 ± 0.61
Idea communication	(4–20)	6.0–18.0	11.64 ± 1.95	2.91 ± 0.49
Implementation starting activities	(3–15)	5.0–15.0	8.29 ± 1.56	2.76 ± 0.52
Involving others	(3–15)	4.0–15.0	8.79 ± 1.70	2.93 ± 0.57
Overcoming obstacles	(4–20)	7.0–19.0	11.27 ± 2.05	2.82 ± 0.51
Innovation outputs	(3–15)	5.0–14.0	8.34 ± 1.52	2.91 ± 0.49
**Overall**	(23–115)	37.0–101.0	66.03 ± 8.35	2.87 ± 0.36

Table 2 presents the descriptive statistics for ambidextrous leadership behaviors and innovative work behavior as perceived by nurses. Overall, nurses reported a moderate level of ambidextrous leadership, with a mean percent score of 40.1 ± 5.7 and an average item score of 2.9 ± 0.4. When broken down by dimension, explorative leadership had a mean percent score of 20.2 ± 3.1, while exploitative leadership scored 19.9 ± 3.4.

Regarding innovative work behavior, the overall mean percent score was 66.0 ± 8.4, with an average item score of 2.9 ± 0.4, also indicating a moderate level of innovation. Among the seven dimensions of the Innovative Behavior Inventory, the highest scores were in idea communication (11.6 ± 2.0) and overcoming obstacles (11.3 ± 2.0). Other dimensions, including idea search (8.9 ± 1.8), idea generation (8.8 ± 1.6), involving others (8.8 ± 1.7), innovation output (8.3 ± 1.5), and implementation of activity (8.3 ± 1.6), also reflected moderate engagement.

**Table 3:** Pearson correlation coefficients between ambidextrous leadership dimensions and innovative work behavior (IWB)

Study variables	Explorative leadership	Exploitative leadership	Overall ALQ
Exploitative leadership	0.554**		
Overall ALQ	0.866**	0.896**	
Idea Generation	0.376**	0.435**	0.348**
Idea Search	0.262**	0.336**	0.342**
Idea Communication	0.254**	0.355**	0.349**
Implementation	0.276**	0.332**	0.346**
Involving Others	0.199**	0.215**	0.235**
Overcoming Obstacles	0.313**	0.362**	0.384**
Innovation Outputs	0.229**	0.277**	0.289**
Overall IBIQ	0.381**	0.466**	0.483**

r = Pearson coefficient *= Statistically significant at *p* ≤ 0.05, ** =Statistically significant at *p* ≤ 0.01

ALO = Ambidextrous Leadership Questionnaire

IBIQ = Innovative Behavior Inventory Questionnaire

**Fig. 3:**
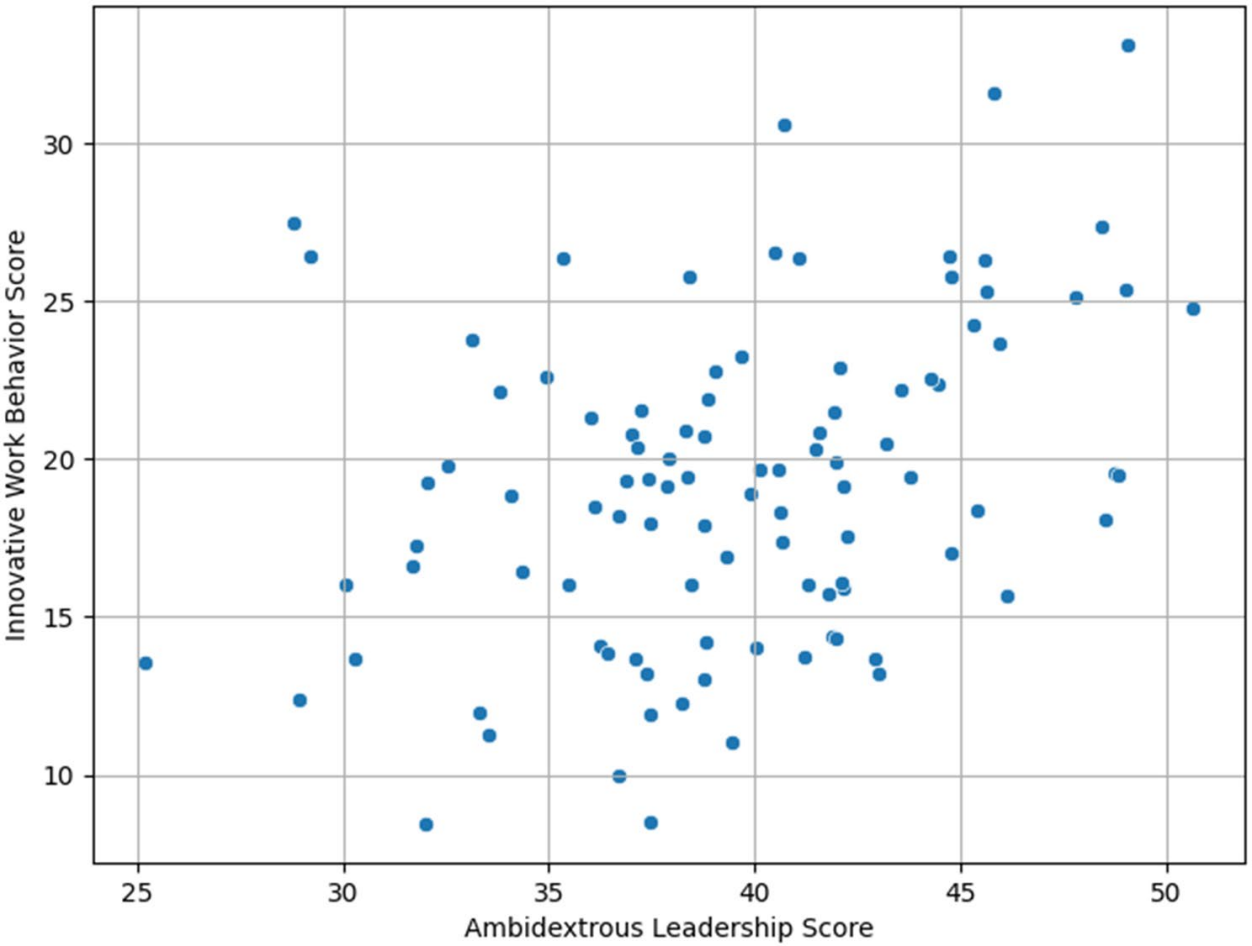
Correlation between ambidextrous leadership and innovative work behavior

Table 3; Fig. 3 show the Pearson correlation coefficients among ambidextrous leadership dimensions and innovative work behavior (IWB). Both explorative and exploitative leadership behaviors exhibited a strong positive correlation with the overall ambidextrous leadership score (*r* = 0.866 and *r* = 0.896, respectively; *p* < 0.001), confirming the relatedness of these constructs. Regarding the innovative work behavior dimensions, all showed significant positive correlations with both types of leadership behaviors and the overall ambidextrous leadership score (all *p* < 0.001).

Notably, exploitative leadership had a stronger correlation with overall IWB (*r* = 0.466) than explorative leadership (*r* = 0.381). The strongest correlations between ambidextrous leadership and specific IWB dimensions were observed for idea communication (*r* = 0.602), implementation starting activities (*r* = 0.762), involving others (*r* = 0.715), and overcoming obstacles (*r* = 0.730), indicating that leadership behaviors significantly influence these key stages of innovation.

**Table 4:** Hierarchical multiple linear regression analysis

Variables	Step 1 (Model 1)					Step 2 (Model 2)				
	B	S.E	Beta	T	*P*	B	S.E	Beta	t	*P*
(Constant)	43.025	9.222		4.666*	< 0.001*	35.757	8.634		4.141*	< 0.001*
Age (years)	1.488	0.156	1.444	9.564*	< 0.001*	1.183	0.150	1.147	7.862*	< 0.001*
Gender [Female]	-0.688	0.788	-0.038	-0.873	0.383	-0.333	0.735	-0.018	-0.453	0.650
Years of experience in the nursing profession	1.346	0.180	1.549	7.496*	< 0.001*	1.070	0.171	1.232	6.254*	< 0.001*
Years of experience in the recent department	0.395	0.108	0.454	3.675*	< 0.001*	0.346	0.100	0.397	3.452*	0.001*
Qualification	2.128	0.644	0.234	3.306*	0.001*	1.533	0.604	0.169	2.536*	0.012*
Marital status [Married]	0.549	0.697	0.034	0.788	0.431	0.652	0.649	0.041	1.005	0.316
Have you ever attended a training program of different leadership styles?	8.519	3.785	0.093	2.251*	0.025*	10.202	3.529	0.111	2.891*	0.004*
Have you ever studied course related to different leadership styles	-1.080	0.915	-0.050	-1.181	0.239	-0.774	0.852	-0.036	-0.909	0.364
Ambidextrous Leadership (ALQ)						0.447	0.060	0.304	7.453*	< 0.001*

R = 0.644, R^2^ = 0.415, F = 31.148^*^, *p* < 0.001^*^ R = 0.704, R^2^ = 0.625, F = 38.163^*^, *p* < 0.001^*^

R^2^ change = 0.210, F = 55.554^*^, *p* < 0.001^*^

**Fig. 4:**
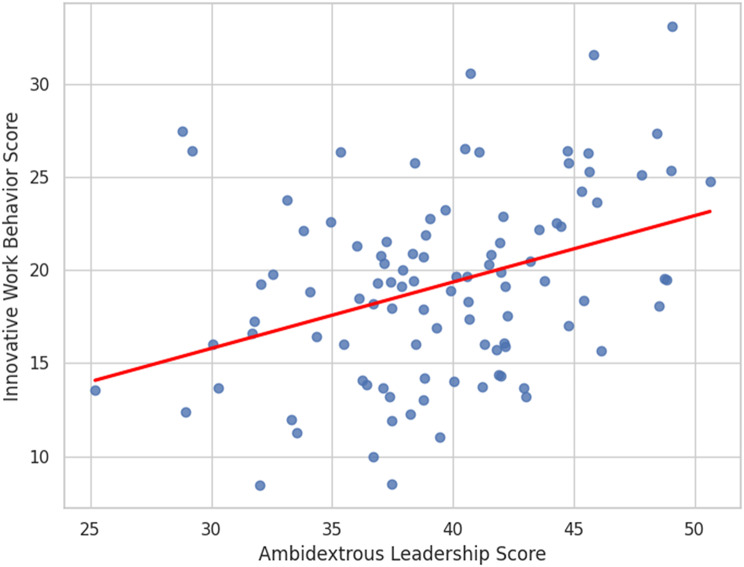
Predictive power of ambidextrous leadership on nurses’ innovative work behavior

Table 4; Fig. 4 present the hierarchical multiple linear regression analysis examining predictors of nurses’ innovative work behavior (IWB). **Model 1** includes demographic and work-related variables. Age (β = 1.444, *p* < 0.001), years of experience in nursing (β = 1.549, *p* < 0.001), years of experience in the current department (β = 0.454, *p* < 0.001), qualification level (β = 0.234, *p* = 0.001), and attendance of leadership style training (β = 0.093, *p* = 0.025) were significant positive predictors of IWB. Gender, marital status, and prior courses related to leadership styles were not significant. This model explained 41.5% of the variance in IWB (R² = 0.415, F = 31.148, *p* < 0.001).

**Model 2** adds ambidextrous leadership (ALQ) as an independent variable. ALQ showed a significant positive effect on IWB (β = 0.304, *p* < 0.001), indicating that higher perceived ambidextrous leadership is associated with increased innovative behaviors among nurses. After adding ALQ, the explained variance increased significantly by 21% (ΔR² = 0.210, *p* < 0.001), with the full model explaining 62.5% of the variance in IWB (R² = 0.625, F = 38.163, *p* < 0.001). The inclusion of ALQ reduced the beta coefficients for some demographic variables, but they mostly remained significant, suggesting ALQ independently contributes to explaining IWB. This means that ambidextrous leadership is a strong, significant predictor of innovative work behavior in critical care nurses, above and beyond demographic and professional factors.

## Discussion

In rapidly evolving healthcare systems, fostering IWB among nurses is increasingly critical for ensuring patient-centered, safe, and high-quality care, particularly in the highly demanding context of critical care environments [16]. These settings are characterized by complex clinical needs, high patient acuity, and time-sensitive decision-making, making innovation both a necessity and a challenge. Leadership plays a decisive role in creating the conditions that either enable or suppress innovation. In this regard, the concept of ambidextrous leadership offers a useful lens for understanding how nurse managers can encourage creativity without compromising reliability and patient safety [14].

The present study revealed that nurses perceived their first-line managers as exhibiting moderate levels of ambidextrous leadership. This finding highlights the structural and cultural realities of critical care units in Egypt, where high workload, resource limitations, and hierarchical organizational structures constrain the consistent application of leadership behaviors [28]. The moderate scores also reflect limited leadership development opportunities within the system, which restrict nurse managers’ capacity to fluidly alternate between exploration and exploitation. Similar patterns were documented by Haider et al. (2023) and Wu et al. (2024), who reported challenges in the consistent application of ambidextrous practices in healthcare [5, 29]. Notably, explorative behaviors scored slightly higher than exploitative ones, perhaps reflecting post-pandemic shifts that have brought innovation, digitalization, and modernization to the forefront of healthcare reform [30–31].

With respect to IWB, nurses reported moderate overall levels, with the strongest performance in idea search and idea generation. This suggests that nurses are willing and able to initiate creative solutions when confronted with challenges. However, scores were lower for idea implementation and overcoming obstacles, signaling that while ideas are generated, systemic barriers—such as rigid protocols, limited autonomy, inadequate resources, and fear of errors—restrict their execution. This pattern mirrors previous studies [32–34], which also observed enthusiasm for idea generation but noted persistent barriers to implementation. In contrast, other investigations (e.g., Fu et al., 2025; Li & Wang, 2025) reported even lower levels of IWB, indicating that cultural and structural conditions strongly shape innovation capacity [35–36]. Our findings therefore add nuance, showing that Egyptian critical care nurses display moderate but uneven engagement in IWB, with organizational and cultural barriers shaping the extent of translation from ideas to action.

The correlational analysis revealed that ambidextrous leadership was significantly and positively associated with IWB, consistent with international evidence linking ambidexterity to enhanced creativity, adaptability, and performance [37–39]. Interestingly, exploitative leadership showed a stronger association with IWB than explorative leadership. This may appear counterintuitive, given that innovation is often assumed to rely primarily on exploration [3]. However, the nature of critical care practice helps explain this finding. These environments are high-stakes and risk-sensitive, where errors have immediate consequences for patient survival. Thus, leadership behaviors that emphasize structure, adherence to standards, and clear guidance may create a sense of psychological safety, allowing nurses to innovate within defined boundaries. This interpretation aligns with studies by Khan & Ullah (2025) and Tho et al. (2025), which highlighted that in contexts of high uncertainty, exploitative leadership provides stability that paradoxically supports innovation [40, 41].

Conversely, the weaker but still positive effect of explorative leadership suggests that while creativity and risk-taking are encouraged, their impact may be moderated by cultural and organizational constraints. Egyptian healthcare institutions are marked by hierarchical authority structures and collectivist norms that value compliance and deference to authority [42]. In such systems, nurses may feel more comfortable pursuing innovative actions when they are accompanied by clear structures and approval from leaders [22]. This may explain why exploitative behaviors had greater influence, while explorative behaviors were present but less impactful. Nevertheless, their positive association suggests an important emerging shift toward modernization, digitalization, and collaborative practices—an encouraging sign in line with global post-pandemic reforms [18, 19].

It is also important to discuss the non-significant findings and variables with weaker associations. Although explorative leadership correlated positively with IWB, its relatively lower effect highlights the contextual limitations of fostering radical or disruptive innovation in critical care. Nurses may experience fear of errors, malpractice stress, or blame within hierarchical environments, which limits their willingness to pursue high-risk innovations [34, 43]. These factors underscore the importance of simultaneously developing psychological safety, supportive policies, and autonomy, alongside leadership training, to maximize the benefits of explorative behaviors.

Our findings also reveal unique contributions when compared with prior research. First, while many studies have emphasized the role of transformational or authentic leadership in nursing innovation [44, 45], our study is among the first in Egypt—and one of the few globally—to empirically examine ambidextrous leadership in critical care nursing. Second, by distinguishing the differential impacts of exploitative and explorative leadership, we highlight that innovation in high-stakes environments is not solely creativity-driven but also discipline-driven. This contributes to leadership theory by demonstrating that exploitative behaviors, often overlooked in innovation studies, can have strong positive effects when contextualized in critical care practice. Finally, our findings show that the Egyptian context—with its hierarchical, resource-constrained, and reform-oriented characteristics—provides unique insights into how ambidexterity operates differently in low- and middle-income countries compared to high-resource settings.

In conclusion, this study provides robust evidence that ambidextrous leadership is a critical driver of innovative work behavior among critical care nurses. The findings demonstrate that while explorative leadership encourages idea generation, exploitative leadership provides the stability and structure that allows ideas to be enacted safely in high-risk environments. This balance is especially relevant in the Egyptian healthcare context, where systemic and cultural factors shape how innovation is perceived and enacted. By integrating ambidextrous leadership into leadership training and organizational strategies, healthcare systems can better equip nurses to drive innovation, improve clinical outcomes, and enhance resilience in the face of ongoing challenges.

### Implications of the study

This study provides meaningful insights into nursing leadership and innovation, particularly in the context of resource-constrained healthcare systems. It advances understanding of how ambidextrous leadership supports IWB among nurses and offers implications for theory, policy, leadership, and clinical practice.

### Implications for nursing leadership and policy

Within the Egyptian healthcare context often challenged by limited resources and hierarchical organizational structures there is a clear need for leadership training programs that cultivate both explorative and exploitative behaviors. Embedding ambidextrous leadership principles into leadership development frameworks can enhance managerial adaptability, promote innovation, and improve care quality in high-pressure environments like critical care units. These efforts align closely with Egypt’s Vision 2030, which emphasizes improving healthcare service quality, fostering innovation, and building a competitive, empowered workforce. Policymakers and healthcare administrators should therefore integrate ambidextrous leadership competencies into national training strategies, link them to performance appraisals and career advancement criteria, and ensure they are tailored to the cultural and operational realities of the Egyptian healthcare system.

### Implications for nursing practice

Findings from the study suggest that ambidextrous leadership significantly influences nurses’ capacity to engage in innovative work behavior. Nurse leaders should aim to create psychologically safe, collaborative environments that encourage team members to share ideas, experiment with new approaches, and solve problems creatively, while maintaining clinical standards. In practical terms, this could involve integrating short innovation-focused huddles into daily rounds, establishing mentorship programs that pair experienced staff with junior nurses to stimulate knowledge sharing, and incorporating innovation goals into individual performance evaluations.

### Implications for research

This study contributes to the growing evidence based on ambidextrous leadership in nursing but also reveals opportunities for further investigation. Future research should explore how leadership interventions targeting ambidexterity influence IWB longitudinally. Comparative studies examining public versus private sector healthcare facilities in Egypt could yield insights into how institutional structures affect the leadership-innovation relationship. Additionally, examining the mediating roles of organizational climate and psychological safety could provide a deeper understanding of the mechanisms through which ambidextrous leadership translates into innovation.

### Limitations of the study

This study has several limitations that should be considered. The cross-sectional design limits the ability to infer causal relationships between ambidextrous leadership and nurses’ innovative work behavior. Additionally, the reliance on a convenience sampling technique may have introduced selection bias and compromised the representativeness of the sample, thereby limiting the external validity and generalizability of the study’s findings. The reliance on self-reported questionnaires also presents a potential for response bias, particularly related to personal perceptions or social desirability. While validated instruments were used, subjective responses may not fully capture actual leadership behaviors or innovation outcomes. Furthermore, the study was conducted in a single tertiary hospital in Egypt, which may limit the applicability of the results to other healthcare settings or cultural contexts.

Future research should consider longitudinal or experimental designs to better establish causality. Expanding the sample to include multiple institutions across varied healthcare contexts would enhance generalizability. Incorporating objective measures of innovation such as peer or supervisor evaluations could reduce bias. Finally, examining contextual factors like organizational culture, team climate, and resource availability may provide a more comprehensive understanding of the conditions that foster innovation in nursing.

## Conclusion

The results of this study revealed a statistically significant positive association between ambidextrous leadership and innovative work behaviour among nurses working in critical care units. Descriptive analyses indicated that both innovative work behaviour and ambidextrous leadership were perceived at moderate levels. Hierarchical multiple linear regression analysis further demonstrated that ambidextrous leadership was a significant predictor of innovative work behaviour, explaining an additional 21% of the variance beyond that accounted for by demographic and professional variables.

## Supplementary Information

Below is the link to the electronic supplementary material.

Supplementary Material 1

## Data Availability

The datasets used and/or analyzed during the current study are available from the corresponding author on reasonable request.

## Abbreviations

IWB: Innovative Work Behavior
ALQ: Ambidextrous Leadership Questionnaire
IBIQ: Innovative Behavior Inventory Questionnaire
CCUs: Critical Care Units
CFA: Confirmatory Factor Analysis
