# Supplementary material for: The impact of ambidextrous leadership on innovative work behavior among critical care nurses: a cross-sectional study
Source: BMC Nurs. 2025 Dec 29;25:94. doi: 10.1186/s12912-025-04232-0 (PMC12849648; doi:10.1186/s12912-025-04232-0)
Supplement: Supplementary file 1 — Supplementary Material 1 [file 12912_2025_4232_MOESM1_ESM.docx]

| **Latent Construct** | **Observed Variable / Subscale** | **Standardized Loading** |
| --- | --- | --- |
| **Explorative Leadership** | ALQ1 – Encourages creativity | 0.82 |
|  | ALQ2 – Supports new ideas | 0.84 |
|  | ALQ3 – Risk-taking | 0.79 |
|  | ALQ4 – Challenges norms | 0.77 |
|  | ALQ5 – Stimulates experimentation | 0.81 |
|  | ALQ6 – Explores alternatives | 0.78 |
|  | ALQ7 – Promotes flexibility | 0.88 |
| **Exploitative Leadership** | ALQ8 – Clarifies expectations | 0.85 |
|  | ALQ9 – Focuses on efficiency | 0.80 |
|  | ALQ10 – Reinforces rules | 0.83 |
|  | ALQ11 – Controls deviations | 0.79 |
|  | ALQ12 – Insists on routines | 0.87 |
|  | ALQ13 – Monitors compliance | 0.66 |
|  | ALQ14 – Stresses discipline | 0.76 |
| **Innovative Work Behavior** | Idea generation | 0.86 |
|  | Idea search | 0.84 |
|  | Idea communication | 0.82 |
|  | Implementation starting activities | 0.70 |
|  | Involving others | 0.72 |
|  | Overcoming obstacles | 0.89 |
|  | Innovation outputs | 0.78 |

**Supplementary Table 1s. Standardized Factor Loadings from the Confirmatory Factor Analysis (CFA) of the Ambidextrous Leadership Questionnaire and the Innovative Behavior Inventory**
